# Metals and Metalloids Release from Orthodontic Elastomeric and Stainless Steel Ligatures: In Vitro Risk Assessment of Human Exposure

**DOI:** 10.1007/s12011-019-01936-8

**Published:** 2019-11-04

**Authors:** Aneta Olszewska, Anetta Hanć, Danuta Barałkiewicz, Piotr Rzymski

**Affiliations:** 1 Department of Facial Malformation, Poznan University of Medical Sciences, Poznań, Poland; 2 Department of Trace Element Analysis by Spectrometry Method, Faculty of Chemistry, Adam Mickiewicz University, Poznań, Poland; 3 Department of Environmental Medicine, Poznan University of Medical Sciences, Poznań, Poland

**Keywords:** Elastomeric ligatures, Metal exposure, Oral cavity, Artificial saliva, Nickel, Orthodontic appliances

## Abstract

Elastomeric ligatures are increasingly used as a part of esthetic orthodontic treatment, particularly in children. The aim of the present study was to experimentally test whether these appliances may contribute to exposure to toxic elements. In the present study, elastomeric ligatures (ELs) were incubated in artificial human saliva for 1 month (a typical period of their use) and the release of 21 metals (Ba, Cd, Co, Cr, Cu, Fe, Li, Mn, Mg, Mo, Ni, Pb, Rb, Tl, Ti, Sb, Sr, Sn, Zn, U, V) and 2 metalloids (As and Ge) was studied using inductively coupled plasma–mass spectrometry. For comparison, stainless steel ligatures (SLs) were incubated for 1, 3, and 6 months (since sometimes their use is prolonged) under similar conditions. The determined metal levels were compared to the corresponding safety limits for human exposure. During 1 month, the ELs released Cd, Co, Cr, Mn, Ni, and Sn at total mean ± SD level of 0.31 ± 0.09, 0.98 ± 0.30, 3.96 ± 1.31, 14.7 ± 8.5, 13.8 ± 4.8, and 49.5 ± 27.7 μg, respectively. Other elements were always below the detection limits. In case of SL, the release of Co, Cr, Fe, Ni, Mn, and Sn was observed, and the determined values increased over the studied period. After 6 months, their total mean ± SD levels amounted to 28.6 ± 0.2, 21.7 ± 0.2, 623.5 ± 3.0, 1152.7 ± 1.8, 5.5 ± 0.3, and 22.6 ± 0.2 μg, respectively. The released metal levels from both ligature types were always below safety limits. The release of Ni from SL during 6 months would constitute 5.0 and 11.5% of tolerable intake in adults and children, respectively. The results of this in vitro study highlight that the use of ligatures in orthodontic treatment can be considered safe in terms of metal exposure although elastic ligatures replaced on a monthly basis appear to be advantageous in comparison to the prolonged use of stainless steel appliances.

## Introduction

The release of trace elements from components installed internally for medical purposes may represent a relevant health threat. For example, elements released from some corrosion-susceptible orthopaedic implant alloys or hip and knee joint replacements can form complexes with proteins, activate the immune system, migrate to different organs, and cause systemic implications [1–4]. It is also well established that amalgamate dental fillings can contribute to exposure to Hg [5], a phenomenon that has eventually led to a ban or a significant restriction on their use in developed countries but has also instilled a fear of poisoning in some dental patients [6]. The use of any orthodontic devices installed intraorally for a prolonged time must ensure that no significant release of toxic ions will occur. Such an assessment is particularly important because of their increased use as a result of improved dental awareness in the general population. The use of orthodontic brackets in particular has become common, and various systems are employed to ligate orthodontic arch wires including stainless steel ligatures and elastomeric ligatures [7].

Elastic ligatures are gaining increasing attention due to a number of advantages of their use: time-efficient, non-difficult application, reduced chair time, better patient comfort and satisfaction, and low cost [8–11]. Several companies manufacture different morphological models (ellipsoid, circular), varying in diameter and width, surface (rough or smooth), and color to meet the esthetical demand of patients and support their cooperation in the treatment process [12–14]. Elastomeric materials used in orthodontics are thermosetting polymers resulting from a step-reaction polymerization of polyesters or glycol polyesters and bi- or poly-isothiocyanates [15, 16]. They are easily pigmented, susceptible to rapid decay of force and deformation during the tensile load applied in the oral cavity, and must be changed periodically, every 3–4 weeks [17, 18]. In turn, stainless steel ligatures are mostly used in the early stages of initial tooth alignment and leveling [19].

Orthodontic appliances and their components are exposed to a variety of intraoral conditions including those of saliva. Their degradation may potentially result in leaching of their components resulting in unintentional human exposure. It is therefore imperative to investigate whether these appliances may release potentially toxic elements during their use and constitute a relevant source of exposure in humans. Some previous studies have already evaluated the release of selected elements for various orthodontic appliances composed of alloys containing Fe, Cr, Ni, Si, and Mo. The in vitro studies have consistently shown that in case of stainless steel appliances, the elements of concern include Ni and Cr [20–24]. This was further confirmed by in vivo observations, indicating that particularly Ni is released to a degree that its increase is detectable in human blood and urine [25–27]. Both, Cr and Ni have been recognized as cytotoxic, mutagenic, and allergenic [28, 29]. At the same time, not much is known on the potential release of elements from elastic ligatures. However, some of these appliances, particularly ligatures composed of latex (which production may involve Zn compounds in the prevulcanization process) and made of polyurethane, were shown experimentally to display toxicity in vitro [30, 31].

To fill this gap, the aim of the present study was to investigate the release of 21 metals (Ba, Cd, Co, Cr, Cu, Fe, Li, Mn, Mg, Mo, Ni, Pb, Rb, Tl, Ti, Sb, Sr, Sn, Zn, U, and V) and 2 metalloids (As and Ge) from elastomeric ligatures varying in color under in vitro conditions of artificial human saliva. Increased and long-term exposure to all of these elements may have an adverse health effects in human encompassing both systemic and organ-specific toxicity [32–34]. The selected ligatures varied in color as this has been previously shown as an important feature determining patients’ preferences, e.g., girls were more likely to choose red and pink ligatures, boys preferred blue and green while adults preferred white and silver [35]. The release of elements from stainless steel ligatures was studied for comparison. Leaching of metals from fixed orthodontic appliances such as brackets or arch-wires has been studied both in vitro and in vivo [20–27] although it is unknown whether this process can also occur in the case of elastic ligatures and pose any relevant health risk. Therefore, the level of metal migration established experimentally in this research was also discussed with reference to maximum safe levels of exposure.

## Material and Methods

### Ligatures

**Fig. 1:**
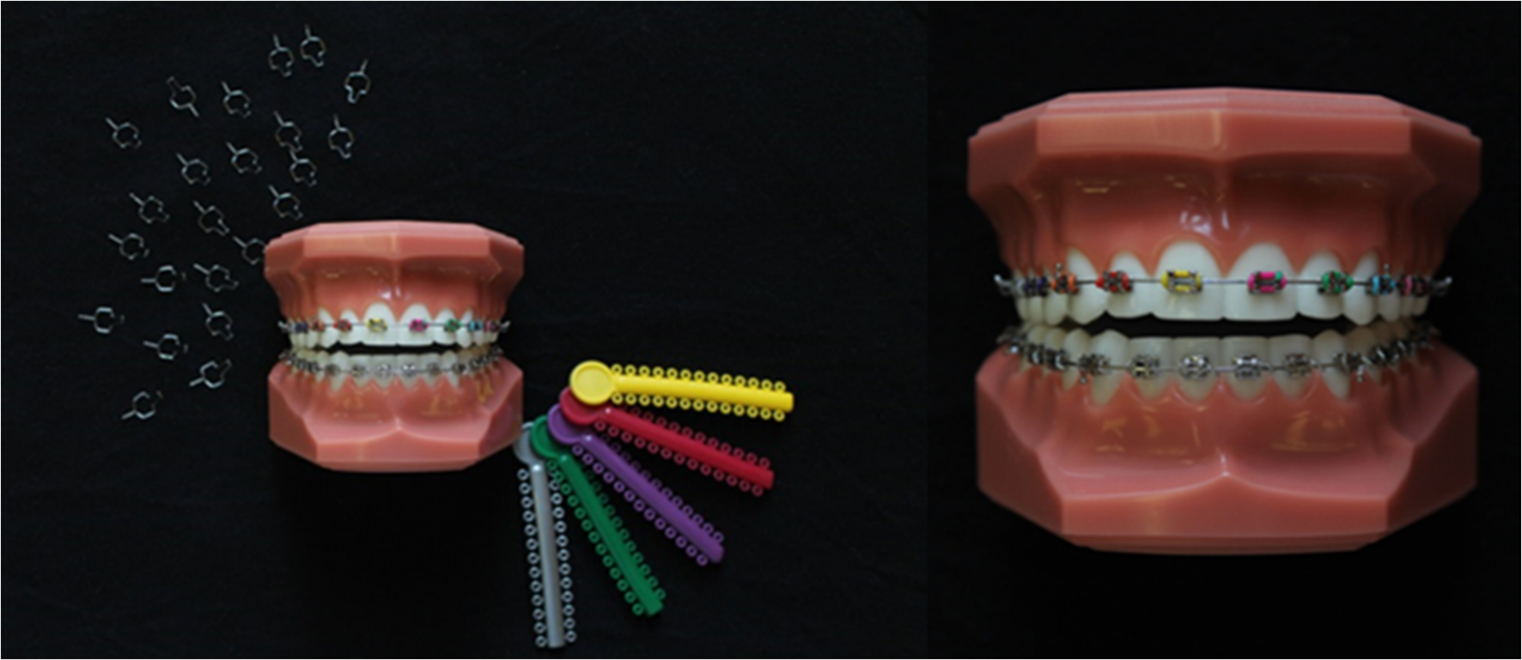
An example of elastic and stainless steel ligatures investigated in the present study

Eleven commercially available latex-free elastomeric ligatures (Fig. 1) made of hypoallergenic medical grade thermoplastic polyurethane were purchased from American Orthodontics (USA), each in a different color: black, blue, burgundy, green, navy blue, orange pink, purple, red, yellow, and white. Stainless steel ligatures were purchased from Ormco (USA). The general composition of the alloy of the stainless steel ligatures was 17–20% Cr, 8–12% Ni, 0.15% C balanced with Fe. All ligatures had recent manufacturing dates at the time of experiment, were unused, and came in sealed plastic packages. The appliances employed in this study are globally distributed, including Europe.

### Experimental Design

The superficial powder coating of the elastomeric ligatures was removed, and all elements were washed for 15 s with deionized water and dried with absorbent paper. Before testing, all ligatures were sterilized by exposure to ultraviolet light (Labconco, USA) for 30 min. All ligatures were immersed in 30 mL of artificial saliva that contained 0.7 g NaCl, 1.2 g KCl, 0.2 g KH_2_PO_4_, 1.5 g NaHCO_3_, 0.26 g Na_2_HPO_3_, and 0.33 g KSCN (Sigma-Aldrich, USA). The elastomeric ligatures were incubated for 1 month (30 days) as this is the usual period of their use. Steel ligatures were incubated for 1 month (30 days), 3 months (90 days), and 6 months (180 days) as they are usually used for a prolonged period of time. All elements were incubated at a constant temperature of 36.6 °C in a thermostatic chamber (Pol-Eko, Poland). After the incubation, saliva was collected and subjected to analytical procedures of multi-elemental analysis following 10-fold dilution. The procedural blanks, consisting of artificial saliva, were prepared in the same way as the other samples without contact with the specimens. Five independent experimental repetitions were performed.

### Trace Element Analysis

The concentration of 23 elements (As, Ba, Cd, Co, Cr, Cu, Fe, Ge, Li, Mn, Mg, Mo, Ni, Pb, Rb, Tl, Ti, Sb, Sr, Sn, Zn, U, and V) in artificial saliva following the incubation with ligatures was determined using an Elan DRC II ICP-QMS (PerkinElmerSCIEX, Ontario, Canada). Samples were introduced to plasma with the use of a cyclonic spray chamber, a concentric glass nebulizer and a quartz torch and injector and Pt cones. Argon was used as a nebulizer, auxiliary, and plasma gas (Linde Gas, Poland). The operating conditions for the ICP-MS were optimized on a daily basis and were as follows: RF power was 1050–1150 W; plasma gas flow rate was 16 L/min; nebulizer gas flow rates were 0.89–0.92 L/min, and auxiliary gas flow rate was 1.2 L/min; lens voltage was 7.00–8.5 V. DRC mode was used in order to eliminate spectral interferences.^52^Cr and ^57^Fe were analyzed in DRC mode with ammonia. A calibration was performed using a multielement stock solution (Multielement Calibration Standard 3, Atomic Spectroscopy Standard, PerkinElmer Pure; Certipur, Merck) containing the analyzed elements at a concentration of 10 mg/L and single element 1000 mg/L Sn solution (Certipur, Merck). A calibration based on a weighed least squares calibration curve was employed for all elements. Analyses were performed using internal standards (ICP Standard CertiPUR, Merck, Germany) to eliminate drift of the instrument and non-spectral interferences. Elements with m/z values that overlap with m/z polyatomic interferents from Ar and matrix components were analyzed in DRC mode. Argon with a purity of 99.999% was used as a nebulizer, auxiliary, and plasma gas for the ICP-MS system (Linde Gas, Poland). High purity ammonia and oxygen were used as DRC reaction gases (Linde Gas, Poland).

For validation of the analytical data applied analytical procedure, two CRMs were used: SLRS-6—river water (NRC, Canada); TM-28.4—fortified lake water (National Water Research Institute, Canada) and by using the analysis of spiked samples. After calibration and also during the analysis, all measurements were controlled by analysis of CRMs. The calibration curves for determined elements were linear in the range of calibration standards. The determination coefficient *R* exceeded a value of 0.999. The details on validation, accuracy and detection limits were previously provided [36]. The limits of detection (LOD) for the determined elements were counted according to LOD = 3.3 *S*/*b*, where *S* means the standard deviation of the result obtained for the blank samples and *b* is the sensitivity (*n* = 5).

### Statistical Analyses and Calculations

Statistical analyses were performed with Statistica 13.0 (StatSoft, USA). Because the data did not meet the assumption of Gaussian distribution (Shapiro-Wilk test, *p* < 0.05), non-parametric methods were applied. The Kruskal-Wallis ANOVA with Dunn’s post hoc test was applied to compare levels of elements released from different elastic ligatures and to compare levels during the different periods of incubation of stainless steel ligatures. The levels of metal release from elastomeric and stainless steel ligatures were compared with the Mann-Whitney *U* test. A *p* value < 0.05 was considered as statistically significant.

To assess human health risks arising from the release of metals from the investigated ligatures, the determined concentrations were confronted with safety intake limits established by the European Food Safety Authority (EFSA) assuming the use of ligatures by 70-kg adults and 30-kg children. These limits are reinforced in European Union although they were established upon the evidence from toxicological studies and as such can serve as a reference point for general estimation of level of exposure to certain elements. Levels of Cd, Cr, and Ni were related to tolerable weekly intake (TWI) set at 2.5, 2.1, and 19.5 μg/kg body weight (bw), respectively [37–39], which in a 70-kg adult and 30-kg children would respectively amount to 175 and 75 μg Cd, 147 and 63 μg Cr, and 1365 and 585 μg Ni. The determined concentration of Mn was related to adequate intake per day as established by the EFSA at a level of 3.0 for adults and 1.5 mg for 10-year-old children [40]. In the case of Fe, the value of the population reference intake set at 11 mg daily was assumed [40]. The determined levels of Co were compared with the health-based guideline level (GL) for chronic exposure for threshold related toxic effects established at a level of 1.6 μg/kg bw per day (equivalent of 112 and 48 μg in a 70-kg adult and 30-kg children, respectively) [41]. For Sn, the PTWI set by the Joint FAO/WHO Expert Committee on Food Additives at a level of 14 mg/kg bw [42] as no regulatory assessment was provided by EFSA. These limits were used in calculation of total safe intake of each element in a period of 30, 60, and 90 days.

## Results

**Fig. 2:**
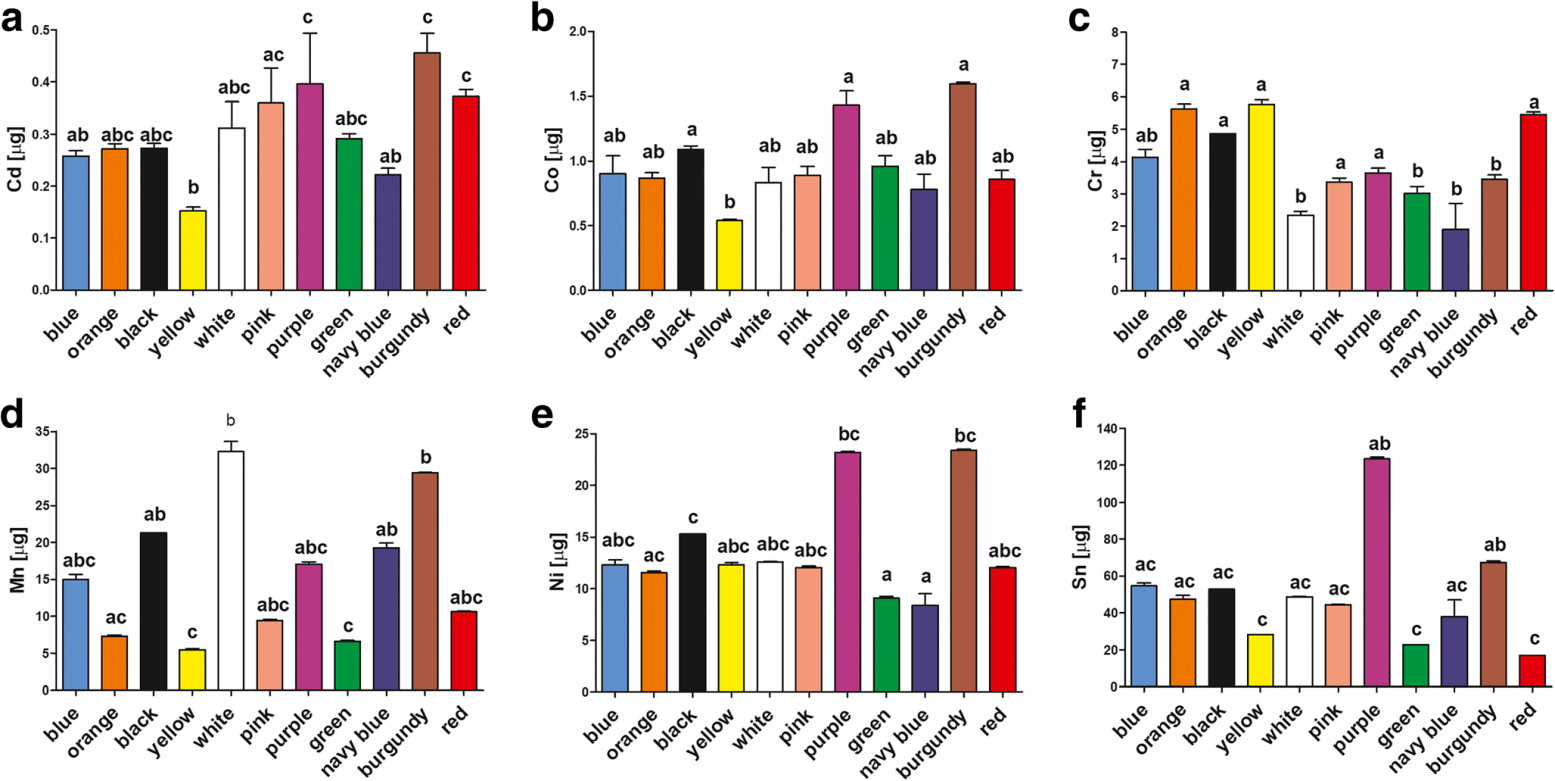
The content (mean ± SD) of metals released from elastic ligatures during 1 month of incubation in artificial saliva. Different superscripts given above columns denote a significant difference between ligatures according to Dunn’s post hoc test (Kruskal-Wallis ANOVA)

Incubation of elastomeric ligatures in artificial saliva for 1 month resulted in the release of Cd, Co, Cr, Mn, Ni, and Sn at a total mean ± SD released level of 0.31 ± 0.09, 0.98 ± 0.30, 3.96 ± 1.31, 14.7 ± 8.5, 13.8 ± 4.8, and 49.5 ± 27.7 μg, respectively. No migration of As, Ba, Cu, Fe, Ge, Pb, Sb, Sc, Tl, and Zn to artificial saliva was observed; the concentrations of these metals were below detection limits. The released levels of metals revealed some differences depending on ligature color (Fig. 2). The highest concentrations of Cd were observed in the case of the burgundy model, Cd, in the case of orange and yellow; Co and Ni, in the case of purple and burgundy; Mn, in the case of white and burgundy; and Sn, in the case of the purple model.

**Fig 3:**
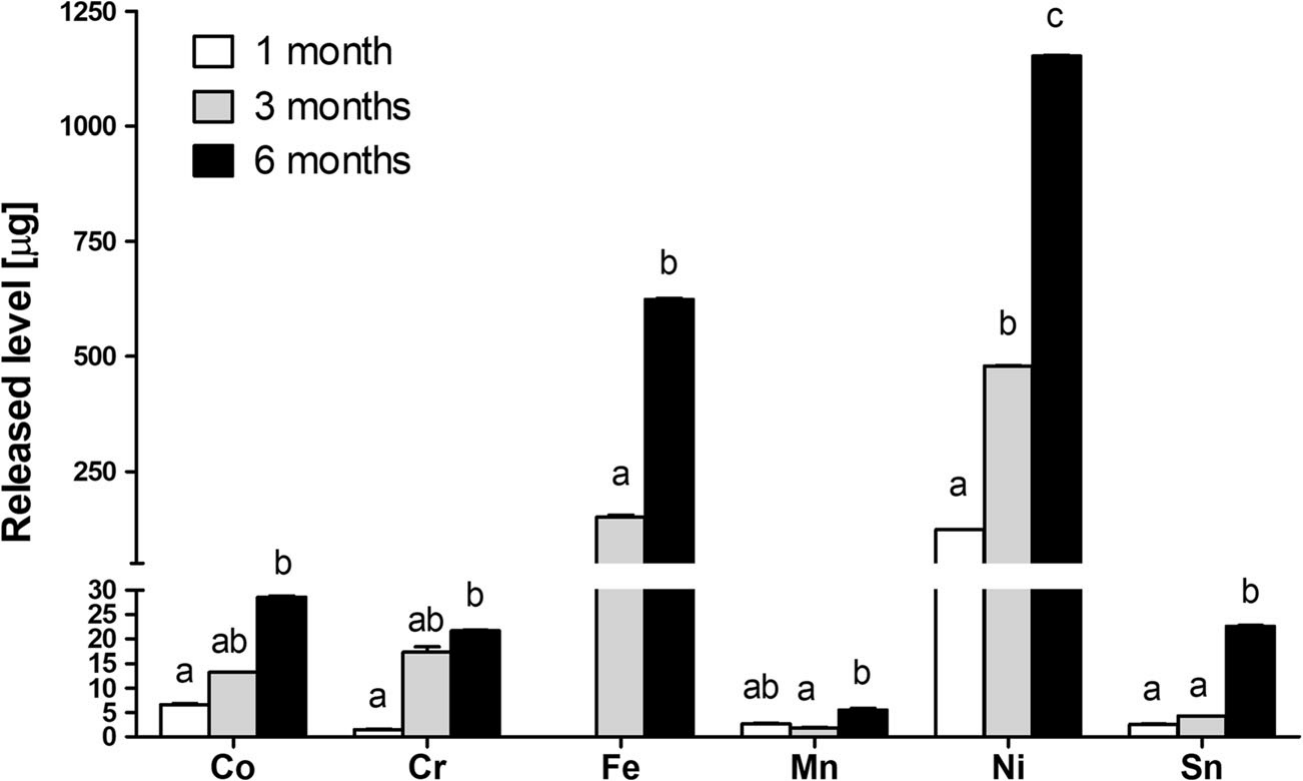
The content (mean ± SD) of metals released from stainless ligatures during 1, 3, and 6 months of incubation in artificial saliva. Different superscripts given above columns denote a significant difference in released elements between the investigated intervals according to Dunn’s post-hoc test (Kruskal-Wallis ANOVA)

The stainless steel ligature released detectable concentrations of Co, Cr, Fe, Ni, and Sn after 1, 3, and 6 months of incubation. Other elements were always below detection limits. The level of released elements increased over the incubation time with the highest concentrations of Co, Cr, Fe, Ni, Mn, and Sn observed after 6 months (Fig. 3). Their total mean ± SD released level at this interval amounted to 28.6 ± 0.2, 21.7 ± 0.2, 623.5 ± 3.0, 1152.7 ± 1.8, 5.5 ± 0.3, and 22.6 ± 0.2 μg, respectively. Compared to contents released from elastic ligatures during 1 month, the stainless steel appliances over the same time interval released 2.5-fold and 6.7-fold higher levels of Ni and Co, respectively (*p* < 0.05 in both cases; Mann-Whitney *U* test), and 9.7-fold, 5.5-fold, and 19.7-fold lower contents of Cr, Mn, and Sn, respectively (*p* < 0.05 in all cases; Mann-Whitney *U* test).

As shown in Table 1, levels of metals released by elastic ligatures during 1 month of incubation were all much below safety limits for adults and children. In the case of stainless steel ligatures, an increase in the contribution to exposure was noted over the experimental period, particularly for Cr and Ni. However, only in the case of the latter element did this contribution exceed 5 and 10% of the safety limit for adults and children, respectively (Table 1).

**Table 1:** Levels of metals released from elastic and stainless steel ligatures in relation to safety limits

Element		Elastic ligatures	Stainless steel ligatures		
		1 month	1 month	3 months	6 months
		% of safety measure^*^ for 70-kg adult/for 30-kg children			
Co	Mean	0.03/0.07	0.2/0.5	0.1/0.3	0.2/0.4
	Max	0.05/0.12	0.2/0.5	0.1/0.3	0.2/0.4
Cd	Mean	0.04/0.01	n.d.	n.d.	n.d.
	Max	0.07/0.18	n.d.	n.d.	n.d.
Cr	Mean	0.7/1.6	0.3/0.6	0.7/1.6	0.9/2.0
	Max	1.0/2.4	0.3/0.7	0.7/1.7	0.9/2.0
Fe	Mean	n.d	n.d	0.01	0.04
	Max	n.d.	n.d.	0.02	0.04
Ni	Mean	0.25/0.59	2.3/5.2	2.9/6.8	5.0/11.5
	Max	0.43/1.0	2.4/5.3	3.0/6.9	5.1/11.6
Mn	Mean	0.01/0.03	< 0.01	< 0.01	< 0.01
	Max	0.03/0.07	< 0.01	< 0.01	< 0.01
Sn	Mean	< 0.01	< 0.01	< 0.01	< 0.01
	Max	< 0.01	< 0.01	< 0.01	< 0.01

*Safety measures used for calculations of total safe intake during 1, 3, and 6 months are explained for each metal in “Material and Methods”

*n.d.* element not detected

## Discussion

The present study demonstrates that the release of trace elements can occur not only from stainless steel orthodontic appliances as previously demonstrated [20–24, 43–46] but also from elastic ligatures. This is most likely due to the chemical degradation of elastomeric elements under wet conditions [47, 48]. However, this phenomenon is limited only to selected metals since the concentration of the majority of the studied elements in artificial saliva incubated with the investigated ligatures were below detection limits. Contrary to stainless steel ligatures, which are sometimes used for a prolonged time, elastic ligatures are recommended to be replaced at every appointment because of force decay and deformation [47, 48]. Therefore, even though the former released lower contents of Cr, Mn, and Sn during the first month, this cannot be considered advantageous. Although differently colored elastic ligatures released varying concentrations of elements, these levels were always low, much below safety guideline values and constituted no threat to the health of adults or children. The present study supports the decision to replace these appliances on a monthly basis not only due to potential changes in their physical properties but also to avoid any relevant exposure of patients to metallic elements during the treatment process. In contrast, the release of metals from stainless steel ligature was demonstrated to increase over the incubation period, and as demonstrated, a relevant content of Ni migrated to saliva during the 6-month period.

As demonstrated, there was no detectable release of As, Ba, Cu, Ge, Pb, Sb, Sc, Tl, and Zn from any studied ligature. From the group of investigated elements, stainless steel appliances released only Co, Cr, Fe, Mn, Ni, and Sn. As previously shown by Mikulewicz et al., the orthodontic appliance consisting of stainless steel wires, bands, brackets, and ligatures may also leach metals such as Mg, Ti, V, Cu, and Zn [21]. Contrary to stainless steel ligatures, their elastomeric counterparts released Cd to artificial saliva. The safety of stainless steel appliances used in orthodontic treatment in this regard has also been confirmed previously [21] and provides a potential advantage of such ligatures compared to the elastomeric ones. The exact source of Cd in elastomeric appliances remains unknown although if one considers the variation of its levels observed between models differing in color, the contribution of dyes used in their production may be hypothesized. The identification of all sources of Cd exposure in humans is of high importance as it can cause systemic organ toxicity and its compounds have been classified as carcinogenic by International Research on Cancer [49]. However, the detected levels in the present study were in magnitudes irrelevant for risk assessment and constitute no threat to human health.

Previously conducted investigations have reported that orthodontic appliances made of stainless steel may release levels of Ni that may pose a risk to human health [20, 21, 50]. The present study confirms these observations and further adds that the Ni content migrating to saliva may increase over the course of ligature use. As observed, patients treated with a self-ligating bracket system made of stainless steel have revealed increased salivary Ni concentrations [51]. This is an important finding since these appliances are fixed in the oral cavity for a prolonged time, often even longer than the studied period. As found, a pool released during 6 months of incubation would constitute 5.0 and 11.5% of tolerable intake for a 70-kg adult and 30-kg children, respectively. These results highlight that the prolonged use of orthodontic stainless steel ligatures may represent a relevant source of Ni intake, particularly important in view of the fact that some individuals can develop a cutaneous reaction after oral Ni intake known as systemic contact dermatitis (SCD) [52]. Cases of SCD following the use of stainless steel orthodontic appliances have been documented [53–55]. Apart from SCD, chronic oral exposure to Ni can lead to other adverse effects, e.g., hematotoxicity and nephrotoxicity [56]. One should, however, note that there are other more relevant sources of oral Ni in the general population, such as selected food supplements or cultivated mushrooms [57–59]. Nevertheless, considering that the content of Ni in selected foodstuffs and subsequent dietary exposures to Ni from food consumption are already of increasing concern [38], an effort should be undertaken to minimize the contribution of additional sources of exposure to this metal. All in all, these observations highlight the advantages of elastic ligatures from which only fractional amounts of Ni, with no relevant contribution to human exposure, were released over the course of 1 month (a typical period of their use).

Other elements which can cause SCD include Cr, Co, and Zn [60, 61]. The release of the latter from dental fillings was shown to be implicated the oral lichen planus, palmoplantar pustulosis, and maculopapular rash [62–64]. No migration of Zn from stainless steel or elastic ligatures was observed in the present study and highlights that these appliances are safe in this regard. One should however note that previous studies have shown that Zn may be released from other stainless steel components of orthodontic appliance (wires, bands, or brackets) [21]. Levels of released Cr and Co from both types of tested ligatures were also too low to constitute any risk for human health in both adults and children, even if prolonged treatment with the stainless steel type would be assumed. This is particularly reassuring if one considers that the composition of the alloy of the stainless steel was composed of 17–20% of Cr. One should also note that the toxicity of Cr strongly depends on its chemical speciation with a hexavalent form considered as a human carcinogen contrary to trivalent compounds which are known to be much less toxic [65]. Stainless steel alloys have in turn been demonstrated to release only the latter forms unless under specific conditions (highly oxidative and alkaline), not present in the oral cavity, are chronically applied [66, 67].

Contrary to stainless steel appliances, none of the investigated elastic ligatures released detectable amounts of Fe. Although the migration from the former increased over the incubation period, the determined concentrations were low and their comparison with the population reference intake indicates that there is no relevant risk of Fe exposure via the use of the appliances in both adults and children. The release of Mn and Sn from elastic and stainless steel ligatures also occurred at a very low level. On comparison with the guideline values, one can conclude that the investigated appliances do not constitute any relevant source of human exposure to these metals. One should also note that Sn is mostly toxic in organic forms, while such compounds are not employed in the production of the orthodontic appliances [68]. Interestingly, the investigated elastic ligatures revealed some variation in the released metals depending on their color. These differences are likely due to the chemical composition of the pigments used during the manufacturing process in order to meet the demands of younger patients, and to prevent color loss during food consumption [69]. In the present study, the most pronounced differences were observed in the case of Sn with a purple ligature releasing at least 2-fold higher levels than other elastic ligatures—this may be due to the use of inorganic Sn (particularly Sn(II) chloride) in purple pigment production [70].

Although the present study provides a general overview on the release of metals from ligatures under mimicked intraoral conditions, one should note that the experiments were conducted under static conditions and that this introduces a study limitation. In the oral cavity, the ligatures are not only exposed to saliva but also to significant and rapid changes in various physicochemical parameters such as pH and temperature, mechanical forces during food consumption, as well as microbial activities that depend on oral hygiene status. All of these can contribute to deformation and degradation of ligatures [69, 71–73] resulting in potentially increased levels of released metals. Moreover, the composition of artificial saliva did not include enzymes which activity may also be relevant for metal release. Therefore, further prospective in vivo studies that would consider the abovementioned parameters are necessary for a full assessment of this phenomenon. One should also note that the subject of this study was a release of metals from orthodontic ligatures not a release from complete dental appliances that also include brackets, arch-wires, retainer alignment, and wire, all of which can be prepared from a variety of materials that may also potentially contribute to metal release. Thus, the use of elastic ligatures instead of stainless steel ligatures may also represent a strategy to decrease to metal exposure in orthodontic patients.

## Conclusions

This study provides an insight into the release of metals and metalloids from orthodontic elastomeric and stainless steel ligatures which are increasingly used in the general population. From the 23 investigated elements, only Co, Cd, Cr, Ni, Mn, and Sn migrated from elastic ligatures to artificial saliva but the observed levels were always much below safety limits. In turn, stainless steel ligatures released Co, Cr, Fe, Ni, and Sn from which only the level of Ni was of concern given the fact that these appliances are used over a prolonged period, not only in adults but also in children. The present study affirms the safety of ligatures in orthodontic treatment, although tending to support the use of elastic ligatures replaced on a monthly basis over the prolonged use of stainless steel counterparts.
